# Liver Kinase B1 Regulates Remodeling of the Tumor Microenvironment in Triple-Negative Breast Cancer

**DOI:** 10.3389/fmolb.2022.847505

**Published:** 2022-06-08

**Authors:** Connor T. King, Margarite D. Matossian, Jonathan J. Savoie, Khoa Nguyen, Maryl K. Wright, C. Ethan Byrne, Steven Elliott, Hope E. Burks, Melyssa R. Bratton, Nicholas C. Pashos, Bruce A. Bunnell, Matthew E. Burow, Bridgette M. Collins-Burow, Elizabeth C. Martin

**Affiliations:** ^1^ Department of Biological and Agricultural Engineering, Louisiana State University, Baton Rouge, LA, United States; ^2^ Department of Medicine, University of Chicago, Chicago, IL, United States; ^3^ Department of Medicine, Section of Hematology & Medical Oncology, Tulane University School of Medicine, New Orleans, LA, United States; ^4^ Xavier University of Louisiana, New Orleans, LA, United States; ^5^ Center for Stem Cell Research and Regenerative Medicine, Tulane University, New Orleans, LA, United States; ^6^ BioAesthetics Corporation, Durham, NC, United States; ^7^ Department of Pharmacology, Tulane University School of Medicine, New Orleans, LA, United States

**Keywords:** LKB1, breast cancer, stem cells, collagen remodeling, tumor micoenvironment

## Abstract

Liver kinase B1 (LKB1) is a potent tumor suppressor that regulates cellular energy balance and metabolism as an upstream kinase of the AMP-activated protein kinase (AMPK) pathway. LKB1 regulates cancer cell invasion and metastasis in multiple cancer types, including breast cancer. In this study, we evaluated LKB1’s role as a regulator of the tumor microenvironment (TME). This was achieved by seeding the MDA-MB-231-LKB1 overexpressing cell line onto adipose and tumor scaffolds, followed by the evaluation of tumor matrix-induced tumorigenesis and metastasis. Results demonstrated that the presence of tumor matrix enhanced tumorigenesis in both MDA-MB-231 and MDA-MB-231-LKB1 cell lines. Metastasis was increased in both MDA-MB-231 and -LKB1 cells seeded on the tumor scaffold. Endpoint analysis of tumor and adipose scaffolds revealed LKB1-mediated tumor microenvironment remodeling as evident through altered matrix protein production. The proteomic analysis determined that LKB1 overexpression preferentially decreased all major and minor fibril collagens (collagens I, III, V, and XI). In addition, proteins observed to be absent in tumor scaffolds in the LKB1 overexpressing cell line included those associated with the adipose matrix (COL6A2) and regulators of adipogenesis (IL17RB and IGFBP4), suggesting a role for LKB1 in tumor-mediated adipogenesis. Histological analysis of MDA-MB-231-LKB1-seeded tumors demonstrated decreased total fibril collagen and indicated decreased stromal cell presence. In accordance with this, *in vitro* condition medium studies demonstrated that the MDA-MB-231-LKB1 secretome inhibited adipogenesis of adipose-derived stem cells. Taken together, these data demonstrate a role for LKB1 in regulating the tumor microenvironment through fibril matrix remodeling and suppression of adipogenesis.

## Introduction

Liver kinase B1 (LKB1) is a serine–threonine kinase that directly phosphorylates proteins in the adenosine monophosphate-activated protein kinase (AMPK) family (Momcilovic and Shackelford 2015). LKB1 has diverse functions such as regulating cell polarity, cell autophagy, and lipid, cholesterol, and glucose metabolism in specialized metabolic tissues (Boudeau et al., 2003; Momcilovic and Shackelford 2015). Loss-of-function and somatic mutations in LKB1 are found in various cancer types, including cervical, ovarian, skin, pancreas, lung, kidney, and breast cancers (Korsse et al., 2013; Nguyen et al., 2021). LKB1 acts as a tumor suppressor in breast cancer to regulate oncogenesis, cancer cell invasion, and metastasis. Inhibition of LKB1 promotes metastasis and invasion in breast cancer and other cancer types (Zhuang et al., 2006; Li et al., 2014), and we have previously reported that LKB1 expression regulates invasive and metastatic properties of basal-like breast cancers (Rhodes et al., 2015). Despite these known functions for LKB1 in invasion and metastasis, the full scope of LKB1 regulatory function of cellular invasion and metastasis is not fully understood, with recent evidence pointing to LKB1 regulation of cellular polarity, matrix remodeling, and cell adhesion. LKB1 utilizes AMPK to modulate cellular polarity and regulate an epithelial phenotype. Loss of this cellular polarity may result in an epithelial-to-mesenchymal transition and increased cellular invasion.

Specific to matrix remodeling, LKB1 inhibits lung cancer progression through lysyl oxidase and extracellular matrix remodeling (Gao et al., 2010a). Another study found that the loss of LKB1 in melanoma disrupts cell migration toward extracellular cues (Chan et al., 2014). In addition, LKB1 modifies cell movement by regulating and suppressing focal adhesion kinase (FAK) (Kline et al., 2013; Konen et al., 2016). LKB1 inhibition also facilitates cellular invasion through collagen matrix realignment, where increased fiber alignments are observed in shLKB1 cell lines (Konen et al., 2016; Lee et al., 2017). These findings demonstrate LKB1’s regulatory mechanism of cancer metastasis through modulation of the extracellular matrix interactions. Furthermore, this implicates LKB1 as a viable target to inhibit breast cancer metastasis and cellular invasion through the modulation of the TME. Targeting proteins interacting with the extracellular matrix has been the focus of many emerging therapeutic investigations, especially in breast cancer systems (Oskarsson 2013; Harisi and Jeney 2015; Venning et al., 2015).

The protein composition and physical properties of the stromal tumor environment provide unique contributions to breast cancer progression. The complex stromal architecture and interactions between the stroma, extracellular matrix, and tumor cells contribute to their invasive and metastatic clinical presentation (Belgodere et al., 2018). For instance, fibril collagen will align to form an angle away from the tumor at the tumor–stroma interface during cancer progression and invasion. The cancerous cells then travel along with this structural formation and invade the surrounding tissues (Provenzano et al., 2006). Furthermore, cancer cells sense mechanical forces within the tissues and through mechano-transduction, alter downstream signaling pathways in response to those forces (Mierke 2014). Kinases such as FAK and PI3K (phosphatidylinositol-3 kinase), actin stress fibers, and focal adhesion cytoskeletal proteins assemble onto the integrin–talin cluster forming a focal adhesion complex. These focal adhesion complexes cross-talk with growth factor receptors and G-protein-coupled receptors, affecting signaling pathways downstream of FAK, such as MAPK (mitogen-activated protein kinase). Focal adhesion complexes use these combined effects, actomyosin contractility and signaling pathways, to alter cellular gene expression and, by extension, cell behavior.^1^ Recent studies are describing cancer cell–stromal interactions as invasion methods, drug resistance, and enhanced cancer stem cell phenotypes (Cazet et al., 2018; Valkenburg et al., 2018; Najafi et al., 2019). Furthermore, we have highlighted that the matrix composition of tumors may promote a pro-tumorigenic scaffold to support cancer growth and invasion (Belgodere et al., 2018). Given current studies demonstrating LKB1 as a regulator of matrix remodeling, we sought to evaluate novel methods of LKB1 regulation of breast cancer metastasis through the evaluation of LKB1-mediated matrix remodeling. Our goal was to investigate the ability of LKB1 to modulate matrix adhesion signaling cascades and responses to a tumorigenic scaffold compared to an adipose scaffold. Furthermore, we evaluated the effects of the tumor scaffold on the matrix and LKB1-associated gene expression.

## Methods

### Reagents

Dulbecco’s modified Eagle’s medium (DMEM), Dulbecco’s phosphate-buffered saline (DPBS), phenol red-free DMEM, fetal bovine serum (FBS), minimal essential amino acids (MEMAA), non-essential amino acids (NEAA), antibiotic/antimitotic penicillin/streptomycin (pen/strep), sodium pyruvate, L-glutamine, trypsin/EDTA, trypan blue stain (0.4%), and ethylenediaminetetraacetic acid (EDTA 0.5 M, pH 8) were obtained from GIBCO (Invitrogen; Carlsbad, CA). Insulin was purchased from Sigma-Aldrich (St. Louis, MO) and charcoal-stripped (CS) FBS from HyClone (Thermo Scientific; Logan, UT). Dimethyl sulfoxide (DMSO) was obtained from Research Organics, Inc. (Cleveland, OH).

### Cell Culture

Human MDA-MB-231 cells were obtained from the American Type Culture Collection (ATCC, Manassas, VA, United States) and are characterized as triple-negative/basal B mammary carcinoma. The MDA-MB-231-LKB1 cell line was generated as previously described (Rhodes et al., 2015). Cells were maintained in DMEM, high glucose (4,500 mg/L) supplemented with 10% FBS Hyclone Cosmic calf serum (HyClone Laboratories, Utah), non-essential amino acids (NEAA), MEM amino acids, anti-anti (100 U/mL), sodium pyruvate, and porcine insulin (1.0 × 10^−10^ mol/L) at 37°C in humidified 5% CO_2._ Human adipose-derived stromal/stem cells (hASCs) were obtained from Obatala (New Orleans, LA). The hASCs were obtained and isolated from lipoaspirate of anonymous female donors and verified for stemness by flow cytometry. The donors were matched for body mass index (average BMI 28.33±0.11) and race (all donors were White), and had an average age of 55.33 years (+/- 18.58 years). The hASCs were maintained in MEM alpha supplemented with 10% Premium Select FBS (Atlanta Biologics), GlutaMax, and pen-strep (Gibco) at 37°C in humidified 5% CO_2_.

### Tissue Collection

The tumor scaffold was derived from the triple-negative patient-derived tumor, previously designated and characterized as TU-BcX-2-K1 (Margarite D. Matossian et al., 2019), and was acquired in collaboration with the Louisiana Cancer Research Consortium Biospecimen Core and processed in compliance with NIH regulations and institutional guidelines and approved by the Institutional Review Board at Tulane University. TU-BcX-2 K1 was established and propagated as previously described (Matossian et al., 2018a). In brief, TU-BcX-2-K1 tumors were grown in immunocompromised SCID/beige mice until a volume of 1000 cm^3^ and then harvested for tumor scaffolds. Human adipose tissue was obtained from the subcutaneous abdominal adipose tissue, and the tissue was obtained from a commercially available source, Obatala Sciences (New Orleans, LA).

### PDX Decellularization

Tumor and adipose scaffolds were generated through the decellularization of PDX tumors and human adipose tissues. Tissues were decellularized through a modified protocol as previously described (M. D. Matossian et al., 2018a; M. D. Matossian et al., 2018b). In brief, tumor samples were collected and left in water for 24 h at 4°C, followed by incubation in Triton X-100 (Bio-Rad, Hercules, CA, Cat. no. 1610406). Cells were first washed in deionized water and incubated in sodium deoxycholate solution (ThermoFisher Scientific, Waltham, MA), then washed in deionized water and incubated in calcium chloride (Sigma-Aldrich, St. Louis, MO), and finally washed with deionized water and treated with DNase I (1 U/ml; Sigma-Aldrich, St. Louis, MO) and antibiotic–antimycotic (100 U/ml; ThermoFisher Scientific, Waltham, MA) solutions.

### Seeding of Tumor and Adipose Scaffolds

Tumor or human adipose scaffolds (5 × 5 mm biopsy punch) were placed in a 24-well plate and covered in DMEM with supplements. The tissues were monitored for signs of contamination for 24 h. Upon verification of a contamination-free scaffold, the scaffolds were then placed into a new 24-well plate without medium. After 5 min, MDA-MB-231- pcDNA vectors or MDA-MB-231-LKB1 cells (2.5 × 10^6^ cells per scaffold) were pipetted onto the scaffolds in a drop-wise fashion. After 15 min of incubation at 37°C in humidified 5% CO_2_, the seeded scaffolds were covered with DMEM. The next day, the seeded tissues were either evaluated *ex vivo* for gene changes or implanted bilaterally into SCID/beige mice (*n* = 3 mice). Tumor growth was measured every 2 days until necropsy. Tumor growth was measured biweekly with digital calipers, and tumor volume was calculated with X and Y dimensions. Volume was approximated using the following formula: 4/3 *π* (L/2)^2 W/2, where L is the longest measurement and W is perpendicular to L. Following necropsy, MDA-MB-231 and LKB1 tumors were removed. Tumor samples were split, with half of each tumor being used for histological analysis and the other half being decellularized and used for proteomics analysis.

### Proteomics

Decellularized tumor and adipose scaffolds (10 mg) were resuspended in 50 μL of 8 M urea and 1 μl of dithiothreitol (500 mM). The samples were shaken at 1,400 rmp for 2 h at 37°C. During alkylation, the samples were then prepared in an iodoacetamide solution in HPLC-grade water. The samples were cooled at room temperature and then 2.5 μl of 500 mM iodoacetamide was added. The samples were then incubated at room temperature in the dark for 30 min. For deglycosylation, 150 μl of 100 mM ammonium bicarbonate, pH 8.0, and 2 μl PNGaseF were added to samples, followed by an incubation period at 1,400 rpm for 2 h at 37°C with continuous agitation. For digestion, 2 μl of a 0.5 μg/μl solution of LysC was added to samples, followed by shaking at 1,400 rpm for 2 h at 37°C. 6 μl of a 0.5 μg/μl solution of trypsin was added to the samples, and the samples were shaken at 1,400 rpm overnight at 37°C. The following day, 3 μl of trypsin was added, and the samples were shaken at 1,400 rpm for 2 h at 37°C. Duging acidification, trypsin was inactivated by adding freshly prepared 50% trifluoroacetic acid (1 μl at a time, pH tested with 1.0 μl of solution onto a pH paper until below 2.0). The sample was then centrifuged at 16,000 x g for 5 min at room temperature. The supernatant was collected and stored at −20°C until further analysis. All samples were desalted, and their protein content was analyzed by using UV–vis spectroscopy at 280 nm. Peptides were labeled using the TMT Isobaric Mass Tagging Kit (Thermo Sci. #90060–90061), according to the manufacturer’s protocol. Protein fractionation and LC-MS/MS analysis were performed on the LTQ-Orbitrap XL instrument (Thermo-Fisher Scientific) coupled to an Ultimate 3000 Dionex nanoflow LC system (Dionex, Sunnyvale, CA).

### Database Search and TMT Quantification

The protein search algorithm used was Mascot v2.3.01 (Matrix Science, Boston, MA). Mascot format files were generated by the Proteome Discoverer 1.2 software (Thermo-Fisher Scientific) using the following criteria: The database, IPI_Human.fasta.v3.77 (containing 89,422 entries and concatenated with the reversed versions of all sequences.); enzyme, trypsin; maximum missed cleavages, two; static modifications, carbamidomethylation (+57 Da), N-terminal TMT6plex (+229 Da), and lysyl TMT6plex (+229 Da). Dynamic modifications, N-terminal Clnpyro-Glu (+17Da); methionine oxidation (+16 Da); STY phosphorylation (+80 Da); precursor mass tolerance was set at 20 ppm; fragment match tolerance was set at 0.8 Da. Peptides reported by the search engine were accepted only if they met the false discovery rate of *p* < 0.05 (target decoy database). A Mascot ion score ≥30 for peptide identifications was required. For TMT quantification, the ratios of TMT reporter ion abundances in MS/MS spectra generated by HCD (up to six reporter ions ranging from *m*/*z* 126.12 to m/z 131.14) from raw data sets were used to calculate fold changes in proteins between control and treatment. All detectable proteins with fold changes are available in Supplementary File S1.

### Conditioned Medium Generation

The secretome of the MDA-MB-231 and MDA-MB-231-LKB1 cells was isolated over 24 h to generate cancer condition medium (CM). Cancer cells were maintained in normal medium until 95–100% confluence. Then, the cells were washed with PBS at target confluence and placed in reduced dextran-stripped 5% FBS phenol-free DMEM. The medium was supplemented with GlutaMax and pen-strep (Gibco). After 24 h, the CM was collected and centrifuged to reduce cellular debris and then stored at −20°C until use.

### Human Adipose-Derived Stromal/Stem Cell Differentiation

Human adipose-derived stromal/stem cells were seeded in full medium and grown until 95–100% confluent. Once confluent, hASCs were washed with PBS and then treated with cancer CM from MDA-MB-231-parental or MDA-MB-231-LKB1 for 3 days. Control hASCs were treated with 5% DMEM or 10% stem medium for 3 days. After 3 days, hASCs were induced for adipogenic differentiation with AdipoQual™ - adipogenic differentiation medium (Obatala, New Orleans, LA) for 7 days. At the endpoint, the cells were fixed with 10% non-buffered formalin and then stained with a solution of Oil Red O (0.3% ORO in isopropyl alcohol and PBS). Phase-contrast images of all samples were obtained on a Nikon Eclipse Ti2 confocal microscope using NIS Elements software. A ×10 objective and a pixel size of ×2048 2048 were used to capture images.

### Statistical Analysis

Studies run in triplicate were analyzed by unpaired Student’s *t-*test (Graph Pad Prism V.4). *p-*values < 0.05 were considered statistically significant. All analyses were run in triplicate. Means are represented in graphs with ±SEM.

## Results

### Tumor Scaffold Enhances Tumorigenesis and Metastasis

We have previously generated a MDA-MB-231-LKB1 cell line and demonstrated that enforced expression of LKB1-decreased breast cancer invasion and metastasis *in vitro* and *in vivo*, respectively (Rhodes et al., 2015). Although there are reports in lung cancer and melanoma models that suggest LKB1 as a regulator of tumor progression and invasion through the remodeling of the tumor matrix, its role in matrix remodeling has yet to be fully explored in breast cancer (Gao et al., 2010b; Chan et al., 2014). To determine the extent to which LKB1 can regulate tumor matrix remodeling and fully identify a role for LKB1–stromal interactions in breast cancer tumorigenesis and metastasis, we utilized a model of tissue decellularization and our previously generated and validated MDA-MB-231-LKB1 overexpressing cell line (Rhodes et al., 2015). To do this, the previously described patient-derived xenograft (PDX) explants (TU-BcX-2-K1, TU-BcX-4QX) (M. D. Matossian et al., 2018a; Margarite D. Matossian et al., 2019; M. D. Matossian et al., 2021) and non-diseased human abdominal adipose tissue were decellularized to obtain tumor and adipose scaffolds, respectively. Tumor and adipose scaffolds were validated for loss of cellular components and matrix structure. Cellular components were identified through H&E in the native tumor. Following decellularization, cellular components were absent in either the adipose or tumor scaffolds (Supplementary Figure S1). Retention of collagen and elastic fibers was evaluated by Mason’s trichrome and Movat’s stains, respectively (Supplementary Figure S1). Results demonstrate loss of cellular bodies and higher collagen and elastic fiber levels in tumor tissue than that in normal adipose tissue.

**FIGURE 1:**
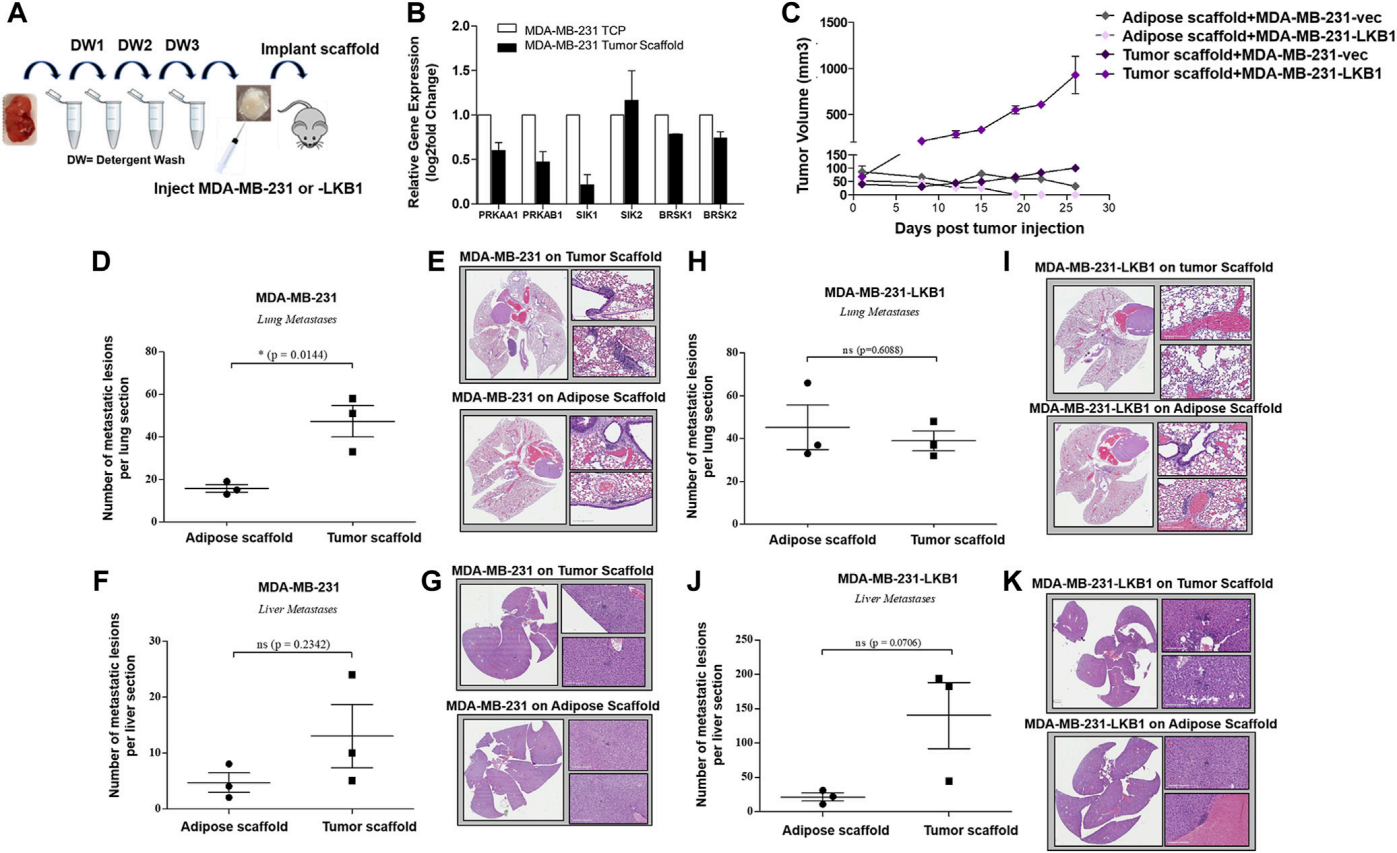
Tumor scaffold enhances tumorigenesis and metastasis. **(A)** Schematic for implantation of decellularized tumor and adipose scaffolds *in vivo*. **(B)** MDA-MB-231 parental cells were seeded on tumor scaffolds for 24 h and evaluated *ex vivo via* qRT-PCR for LKB1- and LKB1-associated genes (PRKAA1, PRKAB1, SIK1, SIK2, BRSK1, and BRSK2). LKB1 was not detected in MDA-MB-231 cells. Normalization was to MDA-MB-231 cells seeded on tissue culture plastic (TCP), housekeeping gene was GAPDH, and error bars represent SEM, *n* = 2. **(C)** Average tumor growth for MDA-MB-231 and MDA-MB-231-LKB1 cells seeded on tumor or adipose scaffolds implanted in the M.F.Ps of SCID/beige mice. **(D–G)** Endpoint necropsy for metastatic lesions in SCID/beige mice implanted in the M.F.Ps with MDA-MB-231 cells seeded on tumor and adipose scaffolds. Results represent **(D)** the number of lung lesions and **(E)** representative H&E images of the lungs, **(F)** number of liver lesions, and **(G)** representative H&E images of the livers. **(H–K)** Endpoint necropsy for metastatic lesions in SCID/beige mice implanted in the M.F.Ps with MDA-MB-231-LKB1 cells seeded on tumor and adipose scaffolds. Results represent **(H)** the number of lung lesions and **(I)** representative H&E images of the lungs, **(J)** number of liver lesions, and **(K)** representative H&E images of the livers. *N* = 3 mice per group for all groups. Organs were harvested at necropsy and were formalin-fixed and paraffin-embedded before staining. DW1-3 = detergent washes 1–3.

Following the confirmation of tumor and adipose scaffold generation (Figure 1A), the role of the tumor architecture and matrix composition on LKB1 signaling was examined. MDA-MB-231 cells were seeded onto tumor scaffolds for 24 h, and gene expression was evaluated *in vitro*. Gene expression changes were compared to MDA-MB-231 cells seeded on tissue culture plastic (TCP). Results demonstrated that the tumor scaffold repressed the AMPK subunits PRKAA1 and PRKAB1. Downstream LKB1 signaling was only modestly altered, as observed through the repression of SIK1. The downstream LKB1 signaling factors SIK2 and BRSK1/2 did not appear to be mediated by the tumor scaffold compared to TCP (Figure 1B).

To determine the role of the tumor architecture and matrix composition on tumorigenesis, MDA-MB-231 cells and MDA-MB-231-LKB1 cells were seeded onto both tumor and adipose scaffolds and then implanted into the mammary fat pads of SCID/beige mice. Implanted scaffolds were measured for tumor growth for 27 days. At the endpoint, both MDA-MB-231 and -LKB1 cells seeded on the adipose scaffold formed smaller tumors compared to MDA-MB-231 and -LKB1 cells seeded on the tumor scaffold (Figure 1C). Furthermore, MDA-MB-231-LKB1-seeded tumor scaffolds had increased tumorigenesis compared to vector seeded tumor scaffolds. To identify a mechanism for the enhanced size of the LKB1 cell line on tumor scaffold compared to the MDA-MB-231 cell line on tumor scaffold, tumor sections were analyzed via Movat’s stain. Histological analysis of tumor and adipose scaffolds suggested that the increased size was due to MDA-MB-231 cell infiltration into the scaffold; MDA-MB-231-LKB1 cells did not infiltrate the scaffolds, and instead, cells remained on the periphery of both the adipose and tumor scaffolds (Supplementary Figures S2A–D). MDA-MB-231 adipose and tumor-seeded scaffolds demonstrated cellular infiltration into the scaffold (Supplementary Figures S2E–H). We next sought to determine if the tumor scaffold altered the metastatic potential of the MDA-MB-231 and LKB1 cell lines. The lungs and liver were histologically evaluated for signs of metastatic lesions. Results demonstrated differences in the metastatic potential that were both scaffold- and cell line-dependent. The tumor scaffold enhanced the formation of metastatic lung lesions in the MDA-MB-231 cell line compared to adipose scaffold-seeded MDA-MB-231 cells (Figures 1D–G). Interestingly, differences in the scaffold type did not result in significant differences in metastasis in the MDA-MB-231-LKB1 cell line (Figures 1H–K).

### LKB1 Regulates Tumor Microenvironment Remodeling

**FIGURE 2:**
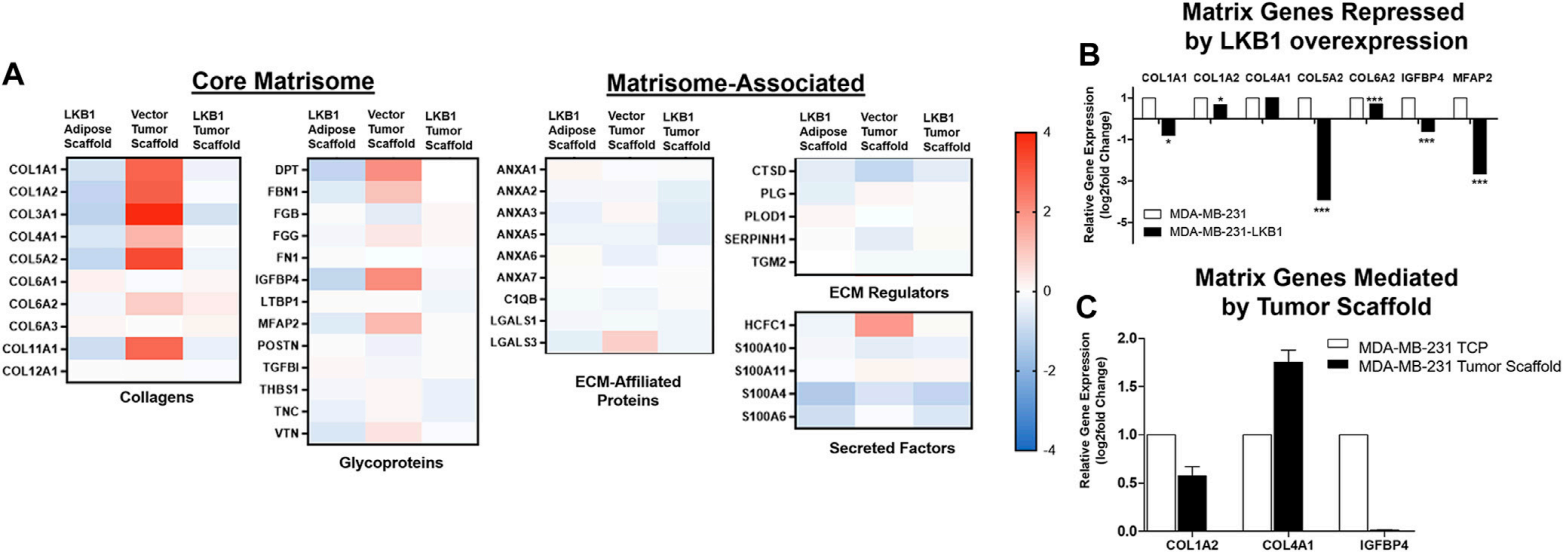
LKB1 overexpression in MDA-MB-231 cells regulates breast tumor matrix remodeling. **(A)** MDA-MB-231 and -LKB1 cells were seeded on tumor and adipose scaffolds and implanted in the M.F.Ps of SCID/beige mice. At necropsy, tumors were removed, decellularized, and evaluated for differences in protein expression through proteomics. Normalization was to MDA-MB-231 cells seeded on the adipose scaffold. Results represent relative fold changes in protein expression for all detected matrix proteins. *N* = 3 mice per group for all groups. Blue bands = decreased protein expression and red bands = increased protein expression. **(B)** Evaluation of previously published RNA sequencing of MDA-MB-231 and MDA-MB-LKB1 (Rhodes et al., 2015) cell lines grown in 2D with a focus on ECM interactions. Results represent fold changes in relative gene expression changes observed in matrix-associated genes in the MDA-MB-LKB1 overexpressing cell line. *p < 0.05, ***p < 0.001. **(C)** MDA-MB-231 parental cells were seeded on tumor scaffolds for 24 h and then evaluated *ex vivo* via qRT-PCR for matrix-associated genes (COL1A2, COL4A1, and IGFBP4). Normalization was to MDA-MB-231 cells seeded on tissue culture plastic (TCP), housekeeping gene was GAPDH, and error bars represent SEM, *n* = 2.

Prior studies in lung cancer and melanoma suggest LKB1 as a regulator of tissue matrix and collagen composition (Chan et al., 2014; Gao et al., 2010a). To determine if MDA-MB-LKB1 cells could remodel matrix composition through the loss of matrix protein expression, proteomics analysis was performed on decellularized tumors derived from MDA-MB-231 and MDA-MB-231-LKB1 cells seeded on tumor and adipose tissue scaffolds. Fold changes in protein expression were compared to MDA-MB-231 cells seeded on adipose scaffolds. Results demonstrated that MDA-MB-231 cells seeded on the tumor scaffold had increased expression of matrix proteins compared to MDA-MB-231 cells seeded on the adipose scaffold. In contrast, both adipose and tumor-seeded MDA-MB-231-LKB1 scaffolds had repression of matrix proteins (Figure 2A; Table 1). Using the Matrisome project (Naba et al., 2012a; Hynes and Naba 2012) (Naba et al., 2012b), the proteomics data were partitioned into protein groups by their function within the ECM. The core matrisome (collagens and glycoproteins) demonstrated the majority of the observed remodeling by LKB1 on the tumor scaffold. MDA-MB-231 cells seeded on the tumor scaffold had an overall increase in the expression of collagen proteins, including major fiber collagens (COL1A1, COL1A2, and COL3A1), fat-related collagen (COL6A2), and glycoproteins (DPT, FBN1, IGFBP4, MFAP2) when compared to MDA-MB-231 cells seeded on the adipose scaffold, while LKB1 had reduced expression of these proteins on both adipose and tumor scaffolds. There were no major changes in matrisome-associated proteins, except for the observed increase in the secreted factor HCFC1 and the ECM-affiliated protein, LGALS3, on the MDA-MB-231-seeded tumor scaffold. Proteomics results suggest LKB1 acts primarily on major fibril collagen and glycoprotein remodeling. Next, previously published next-generation RNA sequencing of the MDA-MB-231-LKB1 cell line was compared to parental MDA-MB-231 cells grown in 2D with a specific focus on matrix and cellular adhesions (Rhodes et al., 2015). Some similarities in repressed matrisome proteins were observed at the transcriptional level. Specifically, LKB1 overexpression resulted in the loss of ECM proteins such as COL1A1, COL5A2, IGFBP4, and MFAP2 (Figure 2B). This suggests that the regulation of ECM may be from both alterations to cancer cells and the TME. To determine if the tumor scaffold alone was able to regulate matrix gene expression, tumor scaffolds were seeded with parental MDA-MB-231 cells for 24 h *in vitro* and collected for matrix gene expression changes. Results demonstrate that tumor scaffold, in the absence of LKB1 overexpression, repressed COL1A2 and IGFBP4, and enhanced COL4A1 compared to MDA-MB-231 cells grown on TCP.

**TABLE 1:** ECM proteins altered with seeding of LKB1 on tumor and adipose scaffold.

Collagens			
Protein	LKB1 AT	Vector 2K1	LKB1 2K1
COL1A1	0.594	7.238	0.826
COL1A2	0.479	7.721	0.951
COL3A1	0.449	15.034	0.594
COL4A1	0.644	2.535	0.980
COL5A2	0.497	9.972	0.836
COL6A1	1.152	0.915	1.098
COL6A2	0.881	1.860	1.250
COL6A3	1.096	1.012	1.125
COL11A1	0.554	6.930	0.779
COL12A1	1.001	1.038	0.958
ECM glycoproteins			
Protein	LKB1 AT	Vector 2K1	LKB1 2K1
DPT	0.478	4.113	0.996
FBN1	0.701	2.132	0.989
FGB	0.936	0.722	1.072
FGG	0.878	1.315	1.071
FN1	1.083	1.047	1.157
IGFBP4	0.490	4.284	0.882
LTBP1	1.037	1.005	0.851
MFAP2	0.709	2.332	1.029
POSTN	0.980	0.828	0.930
TGFBI	1.076	0.893	1.025
THBS1	0.886	1.103	0.811
TNC	0.791	1.090	0.804
VTN	0.655	1.376	0.955
ECM regulators			
Protein	LKB1 AT	Vector 2K1	LKB1 2K1
CTSD	0.716	0.491	0.721
PLG	0.760	1.105	1.018
PLOD1	1.096	0.985	1.041
SERPINH1	1.002	0.730	1.047
TGM2	1.000	0.902	0.897
ECM-affiliated proteins			
Protein	LKB1 AT	Vector 2K1	LKB1 2K1
ANXA1	1.115	0.946	0.934
ANXA2	0.883	0.887	0.761
ANXA3	0.793	1.073	0.708
ANXA5	0.816	0.820	0.686
ANXA6	1.050	0.804	0.918
ANXA7	1.035	0.969	1.011
LGALS1	0.891	0.903	0.850
LGALS3	0.755	1.774	0.840
Secreted factors			
Protein	LKB1 AT	Vector 2K1	LKB1 2K1
HCFC1	0.834	3.610	1.049
S100A10	0.891	0.726	0.800
S100A11	0.913	1.117	1.085
S100A4	0.391	0.626	0.479
S100A6	0.563	0.961	0.657

**FIGURE 3:**
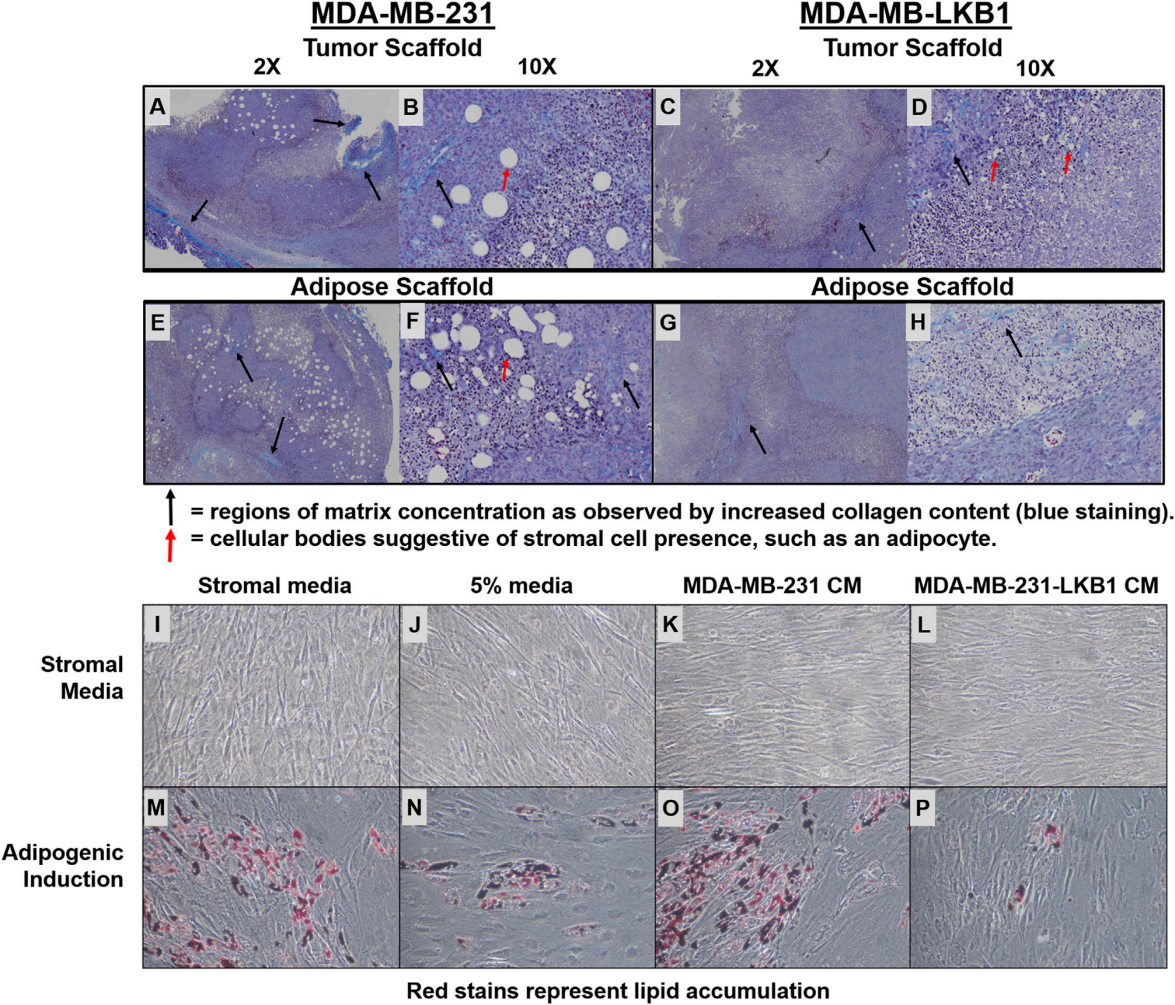
LKB1 overexpression in MDA-MB-231 cells regulates adipose stem cell differentiation. **(A–H)** Confocal microscopy images of Masson’s trichrome (Collagen) staining of **(A–B)** MDA-MB-231 cells seeded on tumor scaffold at ×2 and ×10 magnification, **(C–D)** MDA-MB-231-LKB1 cell-based tumor seeded on tumor scaffold at ×2 and ×10 magnification, **(E–F)** MDA-MB-231 cells seeded on the adipose scaffold at ×2 and ×10 magnification, and **(G–H)** MDA-MB-231-LKB1 cells seeded on the adipose scaffold at ×2 and ×10 magnification. Masson’s trichrome stain: cytoplasm (red), collagen (blue), and nuclei (dark brown). Black arrows highlight regions of matrix concentration as observed by increased matrix staining (blue staining). Red arrows highlight larger cellular bodies suggestive of stromal cell presence, such as an adipocyte. **(I–P)** Phase contrast images of hASCs stimulated with **(I,M)** stromal medium, **(J,H)** 5% medium, **(K,O)** MDA-MB-231-conditioned medium, or **(L,P)** MDA-MB-231-LKB1-conditioned medium for 3 days. Cells were then either maintained in stromal medium **(I–L)** or induced to an adipogenic phenotype with adipogenic medium **(M–P)** for 7 days. Images obtained at ×10 objective. Lipid vacuoles will stain red when present, suggestive of adipogenic differentiation. CM = condition medium.

We next evaluated the fibril collagen content in MDA-MB-231 and -LKB1 cells seeded on tumor and adipose scaffolds to histologically confirm fibril collagen changes. Masson’s trichrome stain was performed on tumor sections obtained from our *in vivo* experiment described earlier. Results demonstrated elevated levels of fibril collagen in MDA-MB-231 cells seeded on both tumor and adipose scaffolds. MDA-MB-LKB1-seeded scaffolds had a markedly decreased expression of fibril collagen, with the loss of collagen being more pronounced in the adipose scaffold than tumor scaffold (Figures 3A–H). In addition to observed collagen remodeling, there was an observed loss of stromal cell infiltration suggestive of a loss in adipocytes. MDA-MB-231 cells seeded on both adipose and tumor scaffolds had observed regions of cells characteristic of adipocytes with lipid vacuoles. In contrast, only MDA-MB-231-LKB1 cells on the tumor scaffold had this observed phenotype. However, suggested lipid size was markedly reduced in LKB1-seeded scaffolds (Figures 3A–H).

### LKB1 Alters Adipogenesis *In Vivo* and *In Vitro*

**FIGURE 4:**
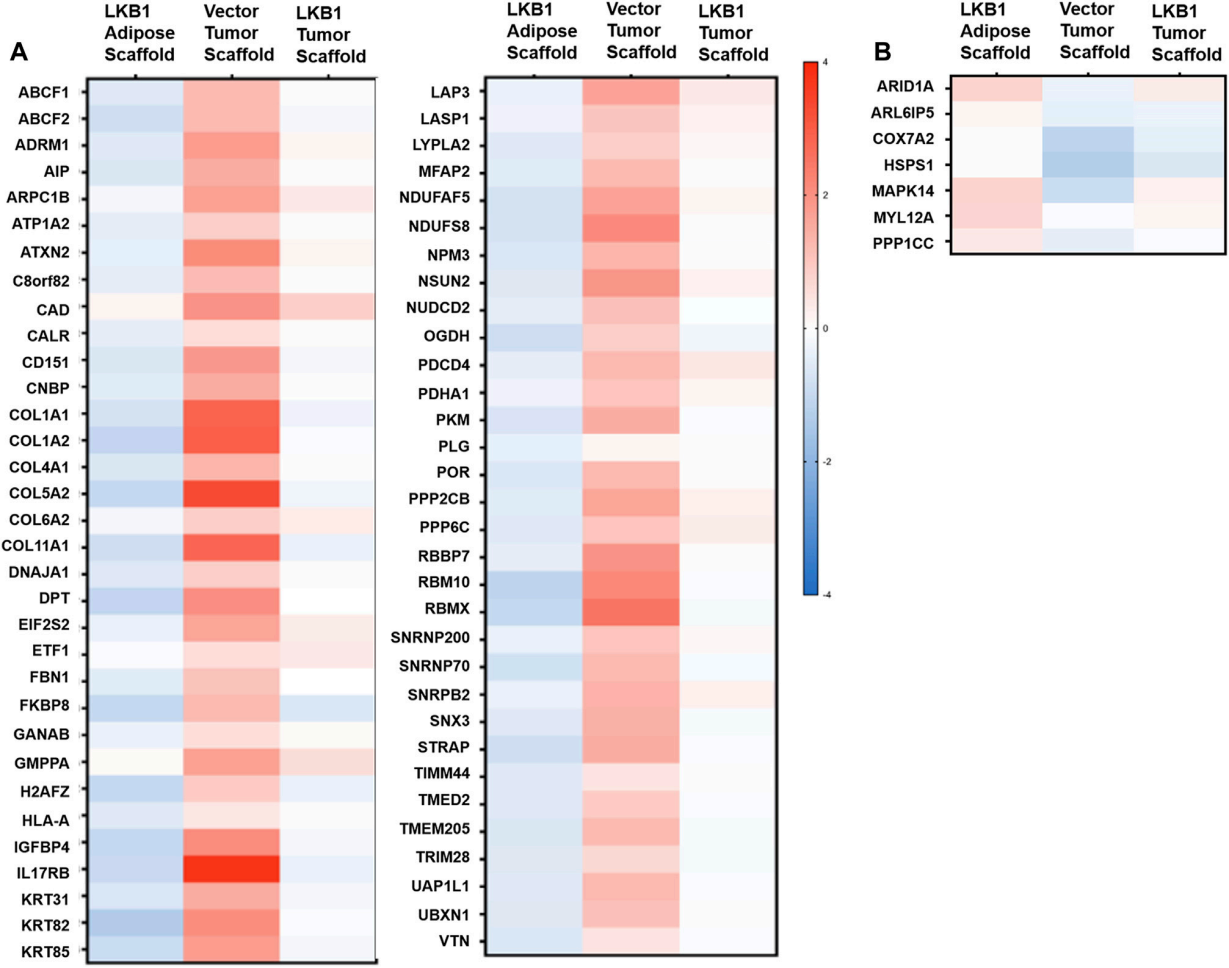
LKB1 overexpression in MDA-MB-231 cells inhibits pro-adipogenic signals in the TME. MDA-MB-231 and LKB1 cells were seeded on tumor and adipose scaffolds and implanted in the M.F.Ps of SCID/beige mice. At necropsy, tumors were removed, decellularized, and evaluated for differences in protein expression through proteomics. Normalization was carried out on MDA-MB-231 cells seeded on the adipose scaffold. Results represent relative fold change in protein expression for all detected proteins that demonstrated an **(A)** increase in the expression in MDA-MB-231 cells seeded on tumor scaffold and repression in protein expression in MDA-MB-231-LKB1 cells seeded on the tumor scaffold or a **(B)** decrease in expression in MDA-MB-231 cells seeded on the tumor scaffold and an increase in protein expression in MDA-MB-231-LBK1 cells seeded on the tumor scaffold. Fold changes in all groups were compared to those in MDA-MB-231 cells seeded on the adipose scaffold. *N* = 3 mice per group for all groups. Blue bands = decreased protein expression and red bands = increased protein expression.

Our histological stains revealed additional differences in TME remodeling between MDA-MB-231- and -LKB1-seeded scaffolds. As stated earlier, large pockets similar to lipid vacuoles were observed within the tumors, suggesting regulation of adipocytes. All highly elevated proteins in MDA-MB-231 cells seeded on tumor scaffolds and repressed by MDA-MB-231-LKB1 on the scaffold were next evaluated (Figure 4). The results demonstrated several ECM-embedded protein regulators of adipogenesis with expression differences between the adipose and tumor scaffold. The expression of positive regulators of adipogenesis (COL6A2, IL7RB, and IGFBP4) (M. Lee et al., 2015; Maridas et al., 2017; Oh et al., 2021) was increased when MDA-MB-231 cells were seeded on the tumor scaffold compared to MDA-MB-231 cells seeded on the adipose scaffolds. Additionally, these factors were repressed in the MDA-MB-231-LKB1 adipose scaffold; however, the tumor scaffold partially rescued this phenotype. The expression of MAPK14, a negative regulator of pre-adipocyte adipogenesis (Qi et al., 2019), was increased when MDA-MB-231-LKB1 was seeded on the adipose and tumor scaffolds (Figure 4).

We next sought to determine if LKB1 expression in cancer cells could regulate adipogenesis in surrounding cells, which was accomplished using a conditioned medium (CM) culture approach. Human adipose-derived stem cells (hASCs) were stimulated for 3.5 days in either MDA-MB-231 CM or MDA-MB-LKB1 CM and then differentiated into an adipogenic phenotype for 7 days. As controls, unstimulated full stromal medium, 5% pretreatment, and unstimulated adipogenic differentiation medium were used. MDA-MB-231 CM treated has maintained adipogenic potential similar to control levels following adipogenic differentiation. However, MDA-MB-LKB1 CM-treated hASCs significantly reduced the adipogenic potential of hASCs (Figure 3C–I, J). These data, taken together with earlier *in vivo* analysis, indicate that LKB1 plays a role in the matrix and stromal remodeling seen within breast tumors.

## Discussion

**FIGURE 5:**
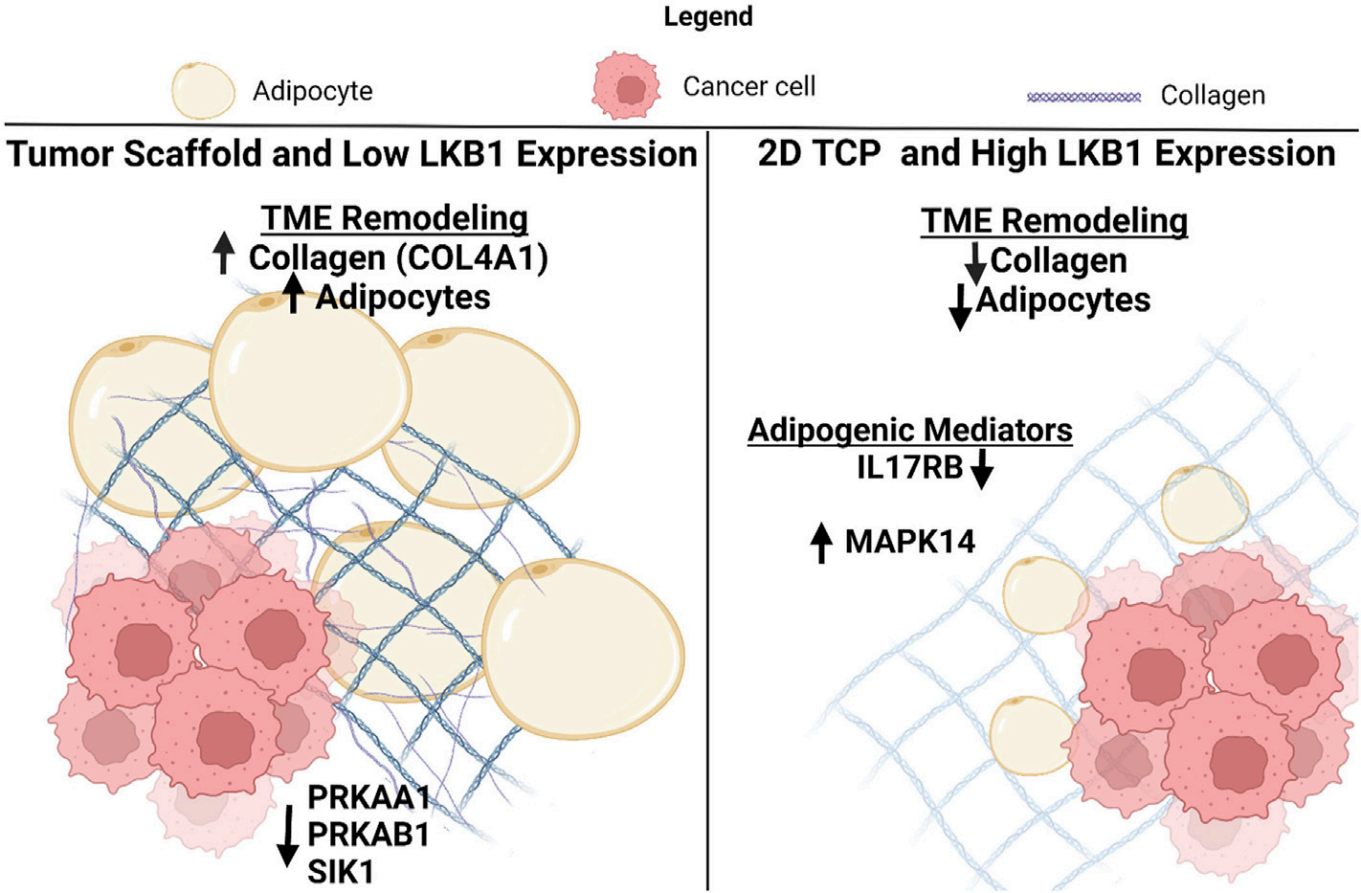
Schematic for the suggested role of LKB1-mediated TME remodeling in TNBC. Created with BioRender.com.

LKB1 is a suppressor of both tumorigenesis and metastasis in breast cancer (Rhodes et al., 2015), but the specific mechanism by which LKB1 suppresses tumor progression remains unclear. A generalized mechanism for LKB1’s role in suppressing tumor progression through the remodeling of the tumor microenvironment is proposed based on the data collected in this study (Figure 5). Specifically, we suggest that LBK1 alters both the matrix and adipogenesis in the TME. Two biological processes that tumors hijack to maintain tumorigenesis were matrix remodeling and organization, which have integral roles in cancer progression and metastasis, resulting in clinically aggressive phenotypes. Understanding the mechanisms of matrix remodeling is integral to identifying the mechanism of cancer progression to a more aggressive phenotype. Tumors increase ECM deposits at the tumor–stromal interface, which promotes increased cell motility and is associated with worse clinical outcomes (Conklin et al., 2011; Riching et al., 2014). Furthermore, increased collagen composition and stiffness are associated with increased drug resistance and cellular proliferation in breast cancer (Liu et al., 2018; Lovitt et al., 2018; Kuczek et al., 2019). Prior studies have utilized decellularized adipose scaffolds to create 3D tumor models (Dunne et al., 2014). Our study is an extension of this and demonstrates the significance of the tumor-specific matrix to drive tumor growth and cell motility. While collagen type I is well described as having a role in cancer cell proliferation, drug resistance, and motility (Barcus et al., 2017; Liu et al., 2018; Lovitt et al., 2018), the matrix proteins such as COL3A1, COL11A1, FBN1, and MFAP2 are less explored in breast cancer. COL3A1, COL11A1, and FBN1 are associated with maintaining a cancer stem-like phenotype in various cancers and therefore may be of interest in breast cancer. To date, MFAP2 has not been evaluated in breast cancer; however, studies in gastric cancer and hepatocellular carcinomas demonstrate that it has elevated expression associated with poor outcomes (Sun et al., 2020; Zhu et al., 2020). Furthermore, repression of MFAP2 results in a loss of cellular proliferation and motility in hepatocellular carcinomas *in vitro* (Zhu et al., 2020).

*In vitro* seeding of tumor scaffolds with MDA-MB-231 cells demonstrated that the tumor scaffold regulated LKB1-associated genes. To date, the effects of tumor-specific scaffold on cancer cell sensing of the TME has not been fully explored, but our studies suggest that the tumor matrix may repress components of the LKB1 signaling cascade. Prior work shows that AMPK and LKB1 mediate autophagy and anchorage-dependent survival through focal adhesions. These recent studies evaluated non-cancer cells and demonstrated mechano-transduction as a positive regulator of AMPK expression. In MCF10 cells and fibroblasts, tension forces and stiff matrix, respectively, increase the expression of AMPK (Bays et al., 2017; Hupfer et al., 2021). Differences between these and the work performed here may be due to the lack of CDH1 in our MDA-MB-231 cell line or due to the comparison of our scaffold to TCP. Furthermore, our study utilized native tumor ECM, while others utilized magnets or artificial scaffolds to induce cell sensing and mechano-transduction.

Unexpectedly, MDA-MB-LKB1 cells seeded on the tumor scaffold were much larger than parental MDA-MB-231 cells on the tumor scaffold. One explanation for this may be that LKB1 cells on both scaffold types maintained growth on the outside of the scaffold, with little motility inward. Prior studies on LKB1 demonstrated LKB1 as an inhibitor of cell invasion and EMT. Specifically, when LKB1 is repressed, there is an observed increase in the expression of EMT transcription factors SNAIL and ZEB1 and subsequent metastasis (Goodwin et al., 2014; Qiu et al., 2018). The association of LKB1 in the regulation of EMT shows the requirement for the loss of LKB1 to invade and leave the tumor. Our seeding model of decellularized adipose and tumor scaffolds placed both MDA-MB-231 and -LKB1 cells on the scaffold periphery and outside of the tissue matrix. Prior studies show that the overexpression of LKB1 inhibits the invasive but not migratory potential of MDA-MB-231 cells (Rhodes et al., 2015). This suggests that the location of our cells on the periphery of the scaffold allowed for metastasis without the need to invade out. Furthermore, as tissue scaffolds would need to be remodeled for cellular motility inward, the loss of cellular invasion in our MDA-MB-LKB1 cell line allowed for the aggregations of cells observed on the periphery of LKB1-seeded tumors compared to MDA-MB-231 cells, which appeared to migrate into the scaffold (Supplementary Figure S2). While prior studies show the need for LKB1 repression to leave the tumor, they did not interrogate LKB1 expression following extravasation. Studies designed to evaluate LKB1 expression in circulating tumor cells have identified a population of circulating tumor cells that stain positive for LKB1 and suggest an oncogenic role for LKB1 in maintaining adhesion-free cellular survival (Trapp et al., 2017). If decellularized scaffolds allow for increased dispersion of cells into the surrounding microenvironment, LKB1 expression may then be advantageous for cell survival and seeding at secondary sites. To fully understand the dynamics of this, additional studies should be conducted that explore the metastatic potential of LKB1-expressing cells following seeding in scaffold cores versus the periphery, in addition to the analysis of LKB1-mediated growth and motility in 3D. Our tumor scaffold had an observed increase in the collagen matrix compared to the adipose scaffold. It is observed by others that increased collagen content and cross-linking lead to increased tumor stiffness (Tang 2020; Martinez-Vidal et al., 2021). This may alter cellular response in terms of cell growth and motility. Many studies demonstrate that stiff scaffolds activate YAP localization and signaling, which is a known target of LKB1 (Qiu et al., 2018; Ondeck et al., 2019; Qin et al., 2020). The full interplay induced by tumor scaffolds, LKB1, and YAP may be an area of interest for understanding how the TME regulates cellular sensing. While not evaluated here, the different effects tumor and adipose scaffold elicit on cell growth and tumorigenesis should be evaluated *in vitro* as well.

Findings from our study demonstrate that LKB1 inhibits matrix gene and protein remodeling (Figure 2), suggesting that LKB1 may repress cancer cell-mediated matrix remolding. Matrix genes observed to be altered in our study have an association with a cancer stem-like phenotype, and LKB1 is associated with the repression of cancer stemness in the lung, gallbladder, and glioblastoma cancers (Caja et al., 2022; Hu et al., 2018; Tong et al., 2021). To date, the role of LKB1 matrix modulation and its role in the repression of cancer stem cell-like phenotype have not yet been interrogated in the study of breast cancer (Nallanthighal et al., 2019).

In addition to matrix remodeling, our results indicate that MDA-MB-231-LKB1 cells release soluble factors that lower the adipogenic potential of hASCs compared to MDA-MB-231 cells (Figure 3). LKB1 regulates the adipogenesis of cells intracellularly by inhibiting early-stage expression of PPARγ and CEBPα in pre-adipocytes (Amélie et al., 2014). Our results expand on this and suggest that LKB1 can regulate the TME through the regulation of adipogenesis. Cancer-associated adipocytes influence breast cancer aggressiveness and provide a pivotal support system to cancer cells in the TME (Wu et al., 2019; Zhao et al., 2020). Furthermore, adipocytes mediate breast cancer cell migration and invasion into the surrounding stroma (Zhao et al., 2020). The findings presented here suggest that LKB1 overexpression negatively regulates adipogenesis within breast tumors. This is observed through LKB1-mediated repression of pro-adipogenesis proteins (IL7RB and IGFBP4) and expression of the adipocyte-associated collagen COL6A2 in scaffolds seeded with MDA-MB-231-LKB1 cells. Adipocyte-associated collagen VI and its cleaved product endotrophin have recently garnered attention as pro-tumorigenic matrix proteins in breast cancer, facilitating cancer cell growth and invasion (Park and Scherer 2012; Karousou et al., 2014; Wishart et al., 2020). Further studies that evaluate the dynamic interplay between LKB1, adipogenesis, and collagen VI matrix production may provide greater insights into adipocyte-mediated tumorigenesis and cancer cell invasion.

These findings provide insights into the utility of tumor-derived scaffolds and LKB1-directed matrix remodeling. Limitations to this study include using a single donor for triple-negative tumor scaffold and adipose scaffold xenograft studies. Furthermore, our study did not normalize for additional tissue factors that may have altered cellular outcomes, such as donor age and BMI. Recent studies have demonstrated that factors such as BMI can enrich matrix genes such as collagen VI and enhance tumorigenesis through matrix composition (Wishart et al., 2020). Future studies should aim to evaluate the effects of LKB1 on multiple TNBC tumor scaffolds compared to adipose scaffolds and should account for matched samples with similar BMI and age. Prior studies have demonstrated the feasibility of decellularized adipose tissue as a 3D tumor model (Dunne et al., 2014). Our initial study is a proof-of-concept study designed to demonstrate the role of LKB1 on TME remodeling and to highlight differences in tumor- and adipose-derived scaffolds. Therefore, the use of multiple donors for tumor and adipose scaffolds was not feasible for this initial study. However, future studies should be performed on additional triple-negative tumors and adipose scaffolds to fully demonstrate the ability of LKB1 to remodel the TME.

## Data Availability

The data presented in the study are deposited in the the Peptide Atlas repository (http://www.peptideatlas.org/), with Dataset Identifier: PASS01763.
