# Supplementary figures and images for: Liver Kinase B1 Regulates Remodeling of the Tumor Microenvironment in Triple-Negative Breast Cancer
Source: Front Mol Biosci. 2022 Jun 8;9:847505. doi: 10.3389/fmolb.2022.847505 (PMC9214958; doi:10.3389/fmolb.2022.847505)

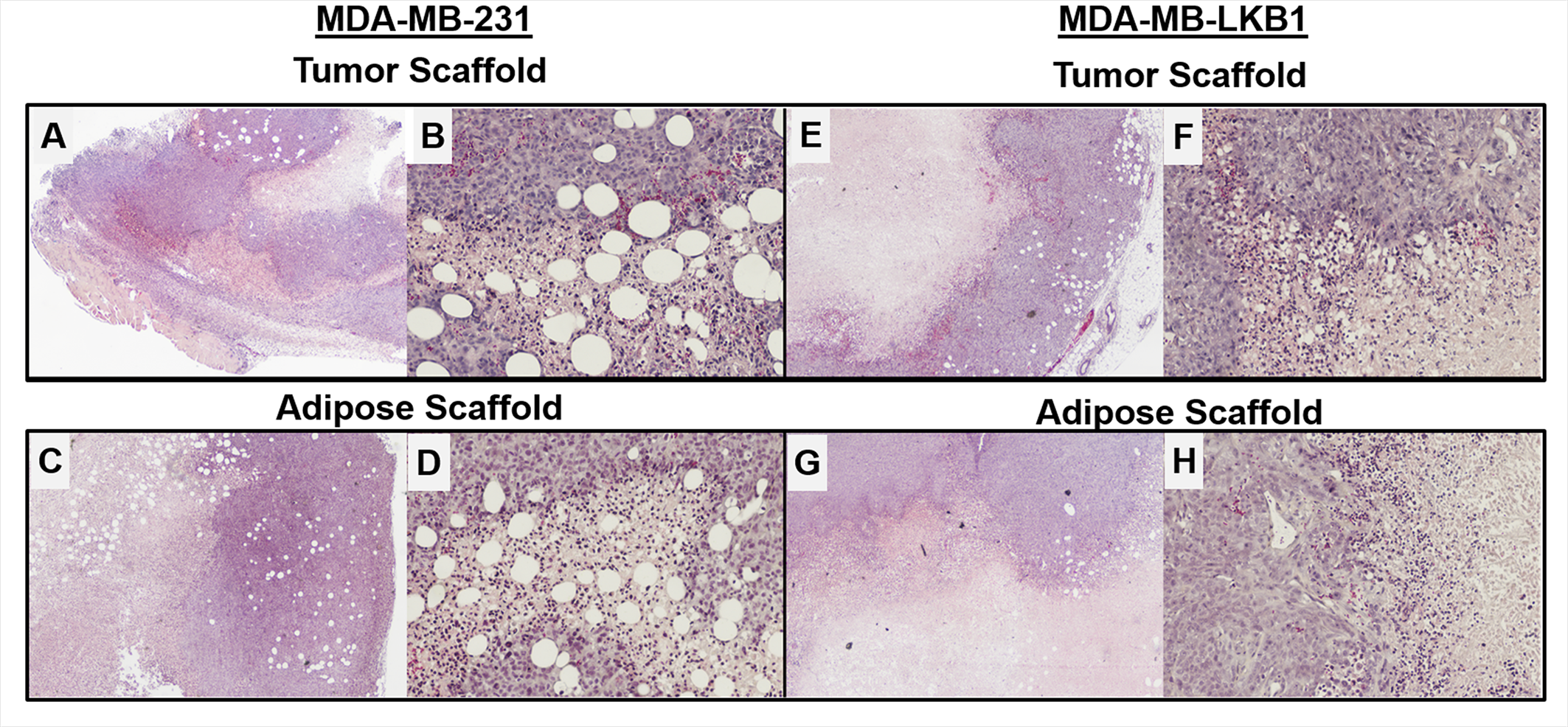

Supplement: Supplementary file 1 [file Image2.TIF]

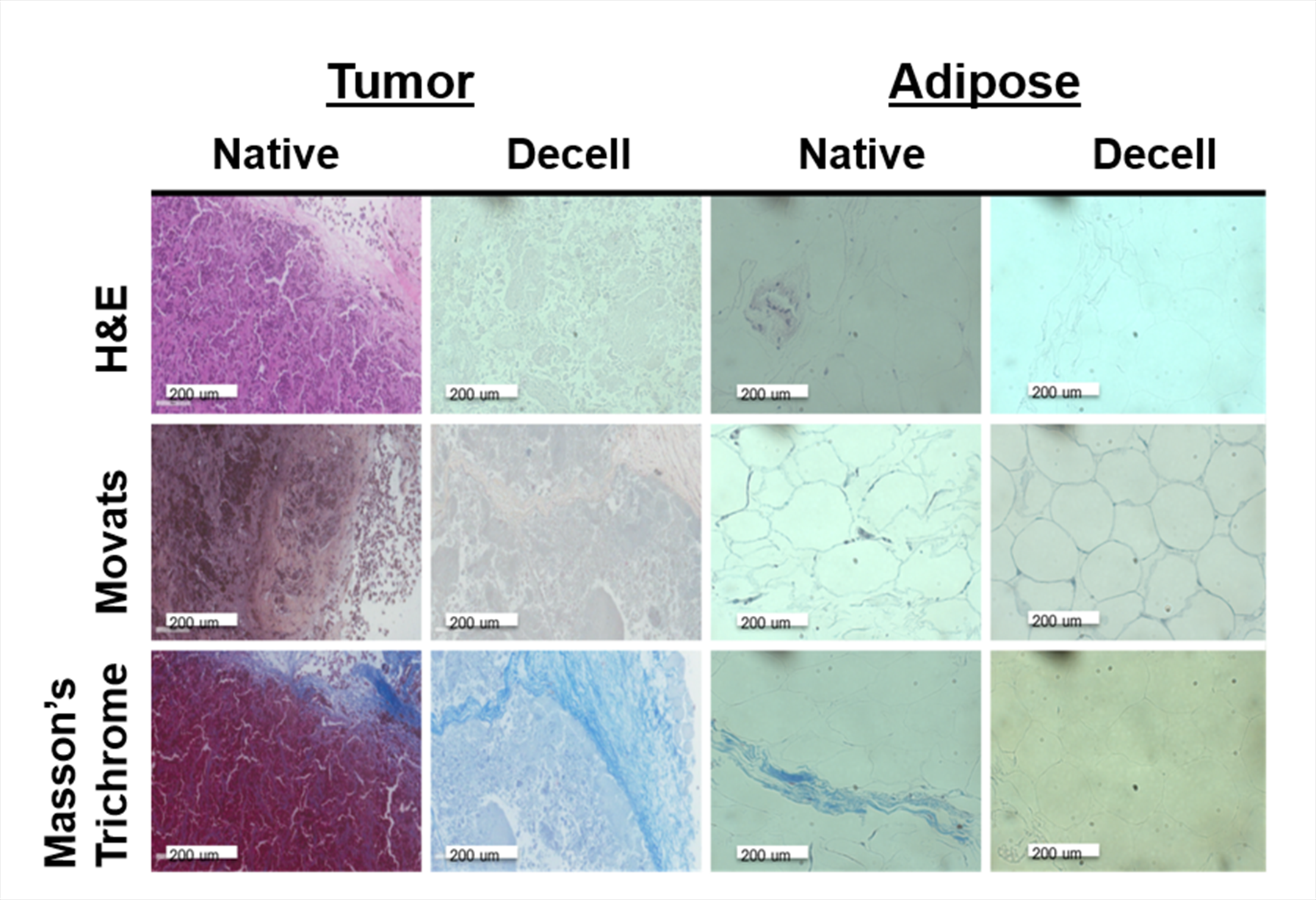

Supplement: Supplementary file 2 [file Image1.TIF]
